# Sex Disparities in Acute Myocardial Infarction Hospitalization, Mortality, and Coronary Angioplasty in the Brazilian Public Health System: A Nationwide Time-Series Study From 2008 to 2024

**DOI:** 10.7759/cureus.117628

**Published:** 2026-10-08

**Authors:** Humberto G Moreira, Yasmin S Moraes, Isadora Rodrigues G Campos, Maria Eduarda Silva F Moraes, Daniel P Coelho

**Affiliations:** 1 Medicine, Universidade Federal de Goiás, Goiânia, BRA; 2 Medicine, Faculdade Morgana Potrich (FAMP), Mineiros, BRA; 3 Medicine, Faculdade da Saúde e Ecologia Humana, Vespasiano, BRA; 4 Medicine, Faculdade Zarns, Itumbiara, BRA

**Keywords:** acute myocardial infarction, brazil, health status disparities, hospital mortality, ischemic heart disease, mortality, myocardial infarction, percutaneous coronary intervention, sex characteristics, sex differences

## Abstract

Background

Women with acute myocardial infarction are treated less invasively than men and die in hospital more often. Whether expanding acute coronary care narrows these differences is unknown.

Methods

We conducted a nationwide time-series study of the Brazilian public health system from 2008 through 2024. Outcomes were hospitalization with acute myocardial infarction (ICD-10 I21-I23) as the principal diagnosis, death with infarction as the underlying cause, and admission for infarction with coronary angioplasty. Rates were standardized by age and sex to the 2022 Census. Trends were estimated by Prais-Winsten regression, and change in the sex disparity was tested as a sex-by-year interaction.

Results

We analyzed 1,927,152 admissions, 1,513,299 deaths, and 543,290 angioplasty admissions. From 2008 to 2024 the age-standardized hospitalization rate rose from 59.8 to 111.7 per 100,000 in men and from 33.8 to 60.0 per 100,000 in women (annual percent change, +3.69% and +3.34%); mortality fell from 69.0 to 54.4 per 100,000 in men and from 46.8 to 34.1 per 100,000 in women (-1.44% and -1.92%, respectively); and angioplasty rose from 11.1 to 41.8 per 100,000 in men and from 5.0 to 20.1 per 100,000 in women (+8.22% and +8.17%, respectively). The men-to-women rate ratio stayed within narrow bands (hospitalization, 1.76 to 1.87; mortality, 1.47 to 1.60; angioplasty, 2.08 to 2.22), and no sex-by-year interaction was significant (p=0.20, p=0.10 and p=0.24). Among admitted patients, standardized in-hospital case fatality fell from 12.9% to 7.0% in men and from 16.1% to 8.3% in women. Women had higher case fatality in every year and every age group from 40 years onward; the adjusted relative risk of in-hospital death for men as compared with women was 0.84 (95% CI, 0.83 to 0.85) and did not change over time (p=0.89).

Conclusions

Brazil's public system expanded admission and angioplasty for myocardial infarction; outcomes improved in both sexes, but the gap between them did not close.

## Introduction

Ischemic heart disease remains the leading cause of death worldwide, and it presents, is recognized, and is treated differently in women and in men. Women are older at presentation, carry more coexisting illness, more often have atypical symptoms or nonobstructive coronary disease, and less often receive invasive and guideline-directed therapy [1,2]. These differences are visible at the scale of whole populations. In Europe and the United States, the ratio of ischemic heart disease mortality to prevalence remains higher in women in several jurisdictions and follows modifiable cardiometabolic and dietary risk [3]; in China, the cardiovascular burden fell faster in women than in men between 1990 and 2016 [4].

Whether such differences narrow as a health system expands its capacity to treat acute coronary syndromes is not known. In pooled trials of primary percutaneous coronary intervention, women had a higher one-year rate of death or hospitalization for heart failure despite similar infarct size and better ejection fraction [5], whereas registries of stable coronary disease and of invasively treated ST-segment elevation infarction report similar adjusted outcomes in the two sexes [6,7]. All of these studies begin at the catheterization laboratory. None can see what happens before it, where recognition, access and delay differ most.

Brazil offers an unusual opportunity to look upstream. Its public Unified Health System serves about three-quarters of a population of more than 200 million [8], records every admission and death in national registries, and has expanded interventional cardiology considerably over the past two decades. The Brazilian evidence is nonetheless fragmented. The decline in ischemic heart disease mortality has stalled or reversed in parts of the country [9]; middle-aged women in one city had excess mortality after infarction [10]; public patients reach a hospital capable of percutaneous intervention nearly three times later than privately insured patients [11]; and national analyses show steep increases in infarction admissions and primary angioplasty from 2008 to 2023, with marked regional inequity [8,12]. No study has followed hospitalization, mortality, and revascularization for the same condition, over the same period, with sex as the axis of comparison.

We examined these three outcomes together over 17 years to determine whether the male excess in each changed as care expanded, and whether the higher in-hospital case fatality of women changed with it. We hypothesized that the gap would narrow, particularly for revascularization.

## Materials and methods

Design and data sources

We conducted a nationwide, population-based ecological time-series study, reported in accordance with the RECORD statement [13]. Hospital data came from the Hospital Information System of the Unified Health System (SIH/SUS) [14], which records every admission reimbursed by the public system, and mortality data from the Mortality Information System (SIM) [15], which covers every death in the country regardless of payer. Diagnoses in both are coded with the International Classification of Diseases, 10th Revision (ICD-10) [16]. We processed the reduced admission files for all 27 federative units and every month of the period; none was missing.

The period, 2008 through 2024, was the same for all three outcomes. It begins when the unified procedure schedule of the public system (SIGTAP), created by Ministry of Health Ordinance 2848 of November 2007, came into force [17]; the codes that identify coronary angioplasty did not exist before it. Because the same transition produced a discontinuity in hospital records, we also tested later start years. The series ends in 2024 because mortality data for 2025 were provisional.

Outcomes

Hospitalization was defined as an admission with ICD-10 code I21, I22, or I23 as the principal diagnosis, and mortality as a death with these codes as the underlying cause. Angioplasty was defined as an admission for infarction, by the same principal-diagnosis criterion, in which one of six SIGTAP percutaneous coronary intervention codes was recorded (0406030014, 0406030022, 0406030030, 0406030049, 0406030065 and 0406030073); angioplasty admissions are therefore a subset of infarction admissions. In-hospital case fatality, the secondary outcome, was the proportion of admitted patients who died before discharge. All ages were included. Admissions were counted as standard (type 1) authorizations; deaths and bed-days also included long-stay (type 5) authorizations, following the convention of the Ministry of Health.

Standardization

Annual denominators by sex and five-year age group were the population projections of the Brazilian Institute of Geography and Statistics (IBGE), 2024 revision [18]. Rates were directly standardized to the population enumerated in the 2022 Census (203,080,756 persons) in 17 age groups [19], with within-sex weights for sex-specific rates and joint age-by-sex weights for both sexes combined. Confidence intervals used the Poisson approximation, and rate ratios were computed on the logarithmic scale. Case fatality was standardized to the age distribution of all admissions for the corresponding outcome over the period, because census weights give undue influence to young age groups with very few admissions.

Statistical analysis

The annual percent change over the whole period was estimated by Prais-Winsten regression of the logarithm of the standardized rate on calendar year, which corrects for first-order autocorrelation. Joinpoint regression, implemented as a grid search with up to two joinpoints, at least four years per segment, and model selection by the Bayesian information criterion, was used descriptively to locate changes in slope.

The primary question, whether the sex disparity changed, was tested as a sex-by-year interaction in a quasi-Poisson model of age-specific counts adjusted for age group, with an F test on the scaled deviance; the same model without the interaction gave the age- and year-adjusted men-to-women rate ratio. We based inference on this test rather than on the annual rate ratios, whose confidence intervals assume Poisson variation and are too narrow for these overdispersed data (scale parameter, 9.9 to 46.6). Age-specific rates were compared between 2008-2012 and 2020-2024.

Six sensitivity analyses were specified in advance, each changing a single input: two alternative standard populations; comparison of standardized with crude rates; a start in 2009 or 2010; exclusion of 2020 and 2021; rescaling of all denominators to the 2022 census count; and, for angioplasty, a broader definition with infarction recorded in any diagnostic field.

Two-sided p-values below 0.05 were considered to indicate significance, without adjustment for multiple comparisons, because the three outcomes address a single question. Records with unknown sex or age were excluded from the corresponding rates. Analyses were performed in Python 3.11, and every reported value was reproduced independently by a verification script. The study used public, aggregated, and anonymized data and was exempt from ethics review under National Health Council Resolution 510/2016.

## Results

Study population

From 2008 through 2024, the public system recorded 1,927,152 admissions for acute myocardial infarction, 1,513,299 deaths from infarction, and 543,290 admissions for infarction with coronary angioplasty (Table 1). Men accounted for 1,223,957 admissions (63.5%), 895,213 deaths (59.2%), and 364,475 angioplasty admissions (67.1%). Women were older at admission (mean age, 64.8 vs. 61.6 years), at death (71.7 vs. 66.6 years), and at angioplasty (64.2 vs. 61.2 years), and about twice as likely as men to be 80 years of age or older in every outcome. Sex and age were missing for no admissions; among deaths, sex was missing for 150 (0.01%) and age for 1403 (0.09%).

**Table 1:** Characteristics of acute myocardial infarction hospitalizations, deaths and coronary angioplasty admissions by sex in Brazil, 2008-2024 Acute myocardial infarction (AMI; ICD-10 I21-I23) [16]. Hospitalization and coronary angioplasty data are from the Hospital Information System of the Brazilian Unified Health System (SIH/SUS) [14]; mortality data are from the Mortality Information System (SIM) [15]. Coronary angioplasty admissions are those with one of six percutaneous coronary intervention codes of the unified SUS procedure schedule (SIGTAP) [17] and AMI as principal diagnosis. *Estimated from five-year age-group midpoints, with 85 years assigned to the open-ended ≥80 group. †Direct standardization by age and sex to the resident population enumerated in the 2022 Brazilian Demographic Census (203,080,756) [19]; annual denominators are IBGE population projections, 2024 revision [18]. Deaths with unknown sex (n=150) and unknown age (n=1403) are included in the mortality totals but excluded from sex-specific and standardized rates, respectively.

Characteristic	Men	Women	Both sexes
AMI hospitalization			
Total admissions, n (% of all)	1,223,957 (63.5)	703,195 (36.5)	1,927,152 (100)
Mean age, years*	61.6	64.8	62.8
<50 years, %	16.1	12.3	14.7
50-64 years, %	42.8	35.6	40.2
65-79 years, %	33.7	38.4	35.4
≥80 years, %	7.4	13.7	9.7
Crude rate per 100,000, annual mean	71.9	39.5	55.3
Age-standardized rate per 100,000, annual mean†	83.1	45.7	63.9
In-hospital deaths, n	113,084	89,101	202,185
In-hospital case fatality, %	9.24	12.67	10.49
Mean length of stay, days	7.15	7.31	7.21
AMI mortality			
Total deaths, n (% of all)	895,213 (59.2)	617,936 (40.8)	1,513,299 (100)
Mean age, years*	66.6	71.7	68.6
<50 years, %	11.6	7.1	9.7
50-64 years, %	30.7	20.5	26.6
65-79 years, %	37.9	37.5	37.7
≥80 years, %	19.8	34.9	26.0
Crude rate per 100,000, annual mean	52.9	34.9	43.7
Age-standardized rate per 100,000, annual mean†	63.9	42.4	52.8
Coronary angioplasty for AMI			
Total admissions, n (% of all)	364,475 (67.1)	178,815 (32.9)	543,290 (100)
Mean age, years*	61.2	64.2	62.2
<50 years, %	15.0	11.3	13.8
50-64 years, %	47.0	39.6	44.6
65-79 years, %	32.6	38.7	34.6
≥80 years, %	5.5	10.4	7.1
Crude rate per 100,000, annual mean	21.3	10.0	15.5
Age-standardized rate per 100,000, annual mean†	24.1	11.3	17.5
In-hospital deaths, n	14,784	10,927	25,711
In-hospital case fatality, %	4.06	6.11	4.73
Mean length of stay, days	4.67	4.89	4.75

Trends in the three outcomes

The three outcomes moved in different directions (Tables 2-3, Figure 1). The standardized hospitalization rate rose from 59.8 to 111.7 per 100,000 in men and from 33.8 to 60.0 per 100,000 in women (annual percent change, +3.69% [95% CI, 3.26 to 4.13] and +3.34% [2.86 to 3.83]), accelerating after 2020 to about 5.4% per year in both sexes; it dipped by only 2.5% in men and 5.0% in women in 2020 and exceeded its 2019 level by 2021. Mortality fell from 69.0 to 54.4 per 100,000 in men and from 46.8 to 34.1 per 100,000 in women (-1.44% [-1.91 to -0.98] and -1.92% [-2.56 to -1.27]); the decline was slow until 2016 and faster thereafter (men, -2.33% per year; women, -3.11%). Angioplasty rose almost fourfold, from 11.1 to 41.8 per 100,000 in men and from 5.0 to 20.1 per 100,000 in women (+8.22% [7.68 to 8.77] and +8.17% [7.64 to 8.70]), growing fastest in the first years and again after 2021. Autocorrelation was modest (coefficients, 0.15 to 0.59), and the Durbin-Watson statistic after correction ranged from 1.60 to 1.92.

**Table 2:** Age-standardized rates and men-to-women rate ratios for the three outcomes, Brazil, selected years Age-standardized rate (ASR) per 100,000 population, directly standardized to the 2022 Brazilian Demographic Census [19]; sex-specific rates use within-sex weights. CI, confidence interval (Poisson approximation). Rate ratios and their confidence intervals were computed on the log scale from the standard errors of the two age-standardized rates and do not account for overdispersion; inference on change over time is based on the sex-by-year interaction.

Outcome and year	Men, ASR (95% CI)	Women, ASR (95% CI)	Absolute difference, men - women	Men-to-women rate ratio (95% CI)
AMI hospitalization				
2008	59.8 (59.2 to 60.3)	33.8 (33.4 to 34.3)	25.9	1.77 (1.74 to 1.80)
2012	72.7 (72.1 to 73.3)	40.7 (40.2 to 41.1)	32	1.79 (1.76 to 1.81)
2016	81.8 (81.2 to 82.4)	45.2 (44.8 to 45.7)	36.6	1.81 (1.79 to 1.83)
2020	89.2 (88.6 to 89.8)	47.6 (47.1 to 48.0)	41.6	1.87 (1.85 to 1.90)
2024	111.7 (111.1 to 112.3)	60.0 (59.5 to 60.5)	51.7	1.86 (1.84 to 1.88)
AMI mortality				
2008	69.0 (68.3 to 69.6)	46.8 (46.3 to 47.3)	22.2	1.47 (1.45 to 1.50)
2012	68.0 (67.4 to 68.7)	46.0 (45.5 to 46.5)	22.1	1.48 (1.46 to 1.50)
2016	67.4 (66.8 to 68.0)	44.8 (44.3 to 45.2)	22.6	1.51 (1.49 to 1.53)
2020	57.8 (57.3 to 58.3)	37.2 (36.8 to 37.6)	20.6	1.55 (1.53 to 1.58)
2024	54.4 (54.0 to 54.9)	34.1 (33.8 to 34.4)	20.3	1.60 (1.58 to 1.62)
Coronary angioplasty for AMI				
2008	11.1 (10.9 to 11.4)	5.0 (4.9 to 5.2)	6.1	2.21 (2.12 to 2.30)
2012	17.0 (16.7 to 17.3)	7.8 (7.6 to 8.0)	9.2	2.17 (2.11 to 2.24)
2016	23.0 (22.7 to 23.3)	10.9 (10.7 to 11.1)	12.1	2.11 (2.06 to 2.17)
2020	30.5 (30.2 to 30.9)	13.8 (13.5 to 14.0)	16.7	2.22 (2.17 to 2.26)
2024	41.8 (41.4 to 42.2)	20.1 (19.8 to 20.4)	21.7	2.08 (2.04 to 2.11)

**Table 3:** Temporal trends in age-standardized rates and in-hospital case fatality by sex in Brazil, 2008-2024 The annual percent change (APC) for the whole period is estimated by a Prais-Winsten regression of the base-ten logarithm of the annual age-standardized rate on calendar year, which corrects first-order autocorrelation (Durbin-Watson after correction 1.60 to 1.92 across series). Segments were identified by joinpoint regression of the logarithm of the annual age-standardized rate (weighted least squares with inverse-variance weights, up to two joinpoints, minimum of four observations per segment, model selected by the Bayesian information criterion); average annual percent change (AAPC) is the length-weighted average of the segment slopes. Joinpoint segments are descriptive; inference rests on the Prais-Winsten estimate and on the interaction test below. ‡Case fatality was standardized to the age distribution of all admissions for the corresponding outcome over 2008-2024. Sex-by-year interaction (age-adjusted quasi-Poisson, F test on the scaled deviance).

Outcome and sex	APC 2008-2024, Prais-Winsten, % (95% CI)	Segment	Segment APC, % (95% CI)	P value	AAPC 2008-2024, % (95% CI)
AMI hospitalization: age-standardized rate					
Men	+3.69 (+3.26 to +4.13)	2008-2020	+3.23 (+2.82 to +3.65)	<0.001	+3.77 (+3.51 to +4.03)
		2020-2024	+5.38 (+4.21 to +6.57)	<0.001	
Women	+3.34 (+2.86 to +3.83)	2008-2020	+2.82 (+2.34 to +3.30)	<0.001	+3.44 (+3.15 to +3.74)
		2020-2024	+5.34 (+4.00 to +6.69)	<0.001	
Both sexes	+3.56 (+3.11 to +4.02)	2008-2020	+3.08 (+2.64 to +3.52)	<0.001	+3.65 (+3.38 to +3.92)
		2020-2024	+5.37 (+4.14 to +6.61)	<0.001	
AMI mortality: age-standardized rate					
Men	-1.44 (-1.91 to -0.98)	2008-2016	-0.52 (-0.95 to -0.09)	0.022	-1.43 (-1.61 to -1.26)
		2016-2024	-2.33 (-2.73 to -1.93)	<0.001	
Women	-1.92 (-2.56 to -1.27)	2008-2016	-0.65 (-1.18 to -0.13)	0.019	-1.89 (-2.10 to -1.68)
		2016-2024	-3.11 (-3.60 to -2.62)	<0.001	
Both sexes	-1.64 (-2.18 to -1.10)	2008-2016	-0.58 (-1.04 to -0.11)	0.018	-1.62 (-1.81 to -1.44)
		2016-2024	-2.65 (-3.08 to -2.22)	<0.001	
Coronary angioplasty for AMI: age-standardized rate					
Men	+8.22 (+7.68 to +8.77)	2008-2014	+9.82 (+9.01 to +10.64)	<0.001	+8.35 (+8.10 to +8.60)
		2014-2021	+6.94 (+6.48 to +7.40)	<0.001	
		2021-2024	+8.74 (+7.70 to +9.79)	<0.001	
Women	+8.17 (+7.64 to +8.70)	2008-2015	+9.30 (+8.31 to +10.29)	<0.001	+8.49 (+8.14 to +8.83)
		2015-2021	+6.47 (+5.69 to +7.26)	<0.001	
		2021-2024	+10.69 (+9.13 to +12.28)	<0.001	
Both sexes	+8.20 (+7.69 to +8.71)	2008-2014	+9.79 (+8.89 to +10.69)	<0.001	+8.41 (+8.14 to +8.68)
		2014-2021	+6.86 (+6.35 to +7.37)	<0.001	
		2021-2024	+9.34 (+8.20 to +10.49)	<0.001	
AMI admissions: in-hospital case fatality‡					
Men	-3.66 (-4.47 to -2.85)	2008-2013	-1.96 (-2.83 to -1.08)	<0.001	-3.74 (-4.00 to -3.49)
		2013-2021	-3.49 (-3.93 to -3.05)	<0.001	
		2021-2024	-7.29 (-8.59 to -5.97)	<0.001	
Women	-3.78 (-4.49 to -3.07)	2008-2021	-3.15 (-3.51 to -2.80)	<0.001	-4.04 (-4.32 to -3.76)
		2021-2024	-7.79 (-9.44 to -6.12)	<0.001	
Both sexes	-3.75 (-4.55 to -2.95)	2008-2013	-2.07 (-2.91 to -1.22)	<0.001	-3.79 (-4.04 to -3.55)
		2013-2021	-3.54 (-3.96 to -3.10)	<0.001	
		2021-2024	-7.27 (-8.54 to -5.97)	<0.001	
Coronary angioplasty: in-hospital case fatality‡					
Men	-3.16 (-3.67 to -2.64)	2008-2021	-2.69 (-3.19 to -2.19)	<0.001	-3.47 (-3.81 to -3.12)
		2021-2024	-6.76 (-8.65 to -4.82)	<0.001	
Women	-2.97 (-3.47 to -2.46)	2008-2021	-2.50 (-3.10 to -1.90)	<0.001	-3.33 (-3.74 to -2.91)
		2021-2024	-6.81 (-9.07 to -4.50)	<0.001	
Both sexes	-3.16 (-3.78 to -2.54)	2008-2021	-2.58 (-2.93 to -2.23)	<0.001	-3.36 (-3.60 to -3.11)
		2021-2024	-6.65 (-8.00 to -5.29)	<0.001	

**Figure 1:**
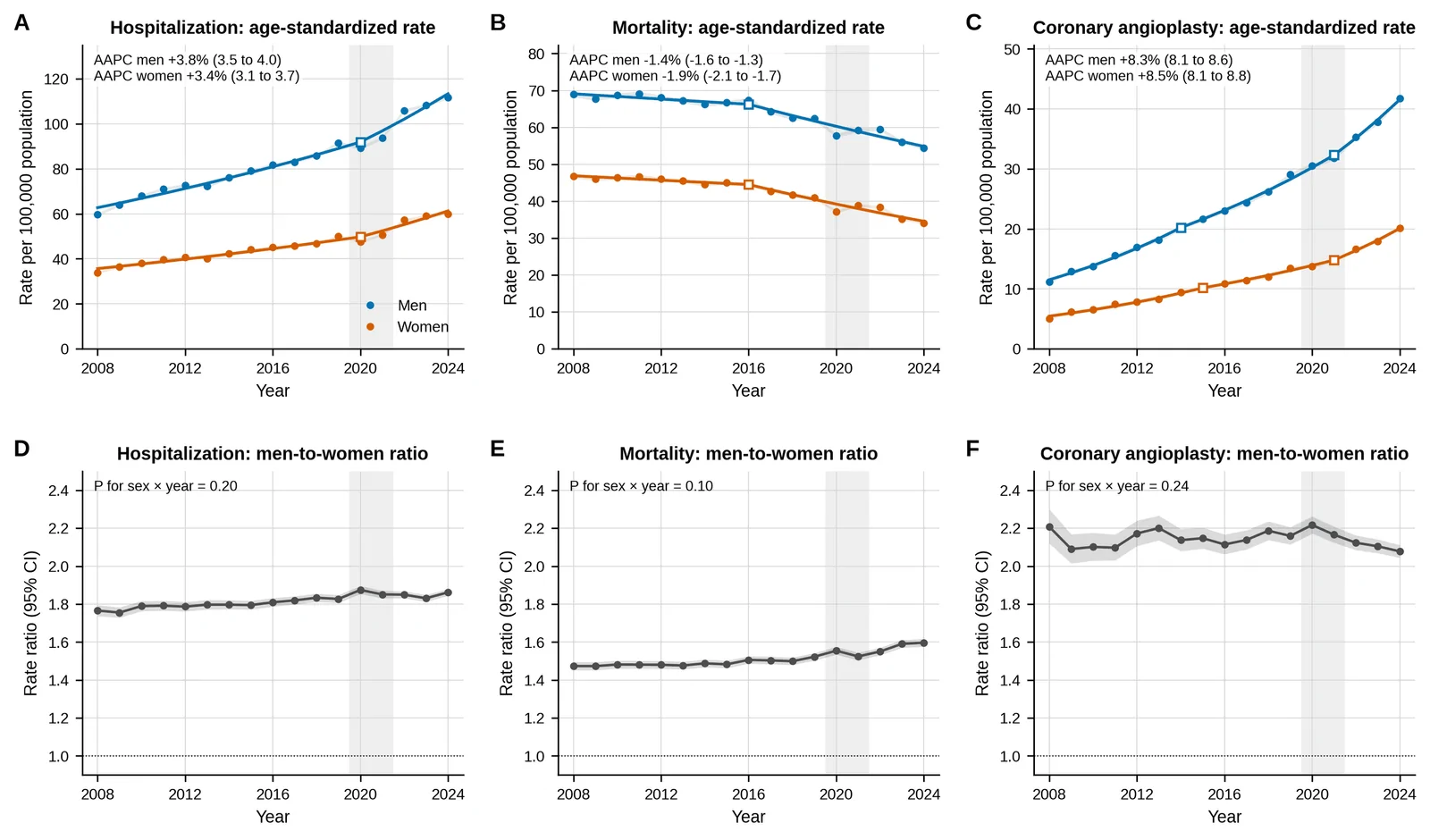
Age-standardized rates and men-to-women rate ratios for acute myocardial infarction hospitalization, mortality and coronary angioplasty in Brazil, 2008-2024 Panels A to C show annual age-standardized rates per 100,000 population in men (blue) and women (orange), with 95% confidence bands, observed values as points, and joinpoint regression fits as solid lines; open squares mark the joinpoints identified. Average annual percent change (AAPC) with 95% confidence interval is inset. Panels D to F show the corresponding annual men-to-women rate ratios with 95% confidence intervals, and the P value of the age-adjusted sex-by-year interaction; the dotted line marks a ratio of 1. The shaded band marks the COVID-19 pandemic years 2020-2021. Rates were standardized by age and sex to the 2022 Brazilian Demographic Census [19]; denominators are the Brazilian Institute of Geography and Statistics (IBGE) population projections, 2024 revision [18]. Sources: Hospital Information System of the Unified Health System (SIH/SUS) [14] for hospitalization and angioplasty; Mortality Information System (SIM) [15] for mortality; International Classification of Diseases, 10th Revision (ICD-10) I21-I23 [16].

The sex disparity

Through these divergent trajectories, the men-to-women rate ratio barely moved (Table 2, Figure 1). It ranged from 1.76 to 1.87 for hospitalization, from 1.47 to 1.60 for mortality, and from 2.08 to 2.22 for angioplasty. No sex-by-year interaction was significant: the difference between men and women in annual percent change was 0.22 percentage points (95% CI, -0.11 to 0.55; p=0.20) for hospitalization, 0.38 (-0.07 to 0.84; p=0.10) for mortality, and -0.24 (-0.63 to 0.16; p=0.24) for angioplasty. These intervals exclude any change in the disparity larger than about 0.6 to 0.8 percentage points per year. Over the whole period, the adjusted men-to-women rate ratio was 2.09 (95% CI, 2.05 to 2.12) for hospitalization, 1.85 (1.81 to 1.90) for mortality, and 2.42 (2.38 to 2.47) for angioplasty. Because rates rose while ratios held, the absolute excess in men doubled for hospitalization (from 25.9 to 51.7 per 100,000) and more than tripled for angioplasty (from 6.1 to 21.7 per 100,000), whereas for mortality it changed little (from 22.2 to 20.3 per 100,000).

The crude proportion of infarction admissions that included angioplasty doubled in both sexes, from 7441 of 39,832 (18.7%) to 43,399 of 116,097 (37.4%) in men and from 3435 of 23,050 (14.9%) to 22,280 of 66,479 (33.5%) in women. The gap between them ranged from 3.0 to 5.3 percentage points across the period and was 3.9 points in 2024, close to its 2008 value of 3.8.

The male excess was greatest in early and middle adulthood and narrowed with age in every outcome (Table 4, Figure 2). In 2020-2024, the rate ratio fell from 2.96 at 30 to 34 years to 1.57 at 80 years or older for hospitalization, from 3.11 to 1.36 for mortality, and from 3.26 to 1.75 for angioplasty. Between 2008-2012 and 2020-2024 the mortality ratio drifted upward at most ages above 40 years (at 50 to 54 years, from 2.49 to 2.74); the other ratios were essentially unchanged.

**Table 4:** Age-specific rates and men-to-women rate ratios for the three outcomes in Brazil, 2020-2024 Rates are events per 100,000 population per year, pooled over 2020-2024. Age groups below 20 years contributed few events and are omitted; the corresponding values for 2008-2012 are shown in Figure 2. Denominators are the Brazilian Institute of Geography and Statistics (IBGE) population projections, 2024 revision [18]. RR - men-to-women rate ratio; CI - confidence interval (Poisson approximation); AMI - acute myocardial infarction

Age group (years)	AMI hospitalization: men	AMI hospitalization: women	AMI hospitalization: RR (95% CI)	AMI mortality: men	AMI mortality: women	AMI mortality: RR (95% CI)	Coronary angioplasty for AMI: men	Coronary angioplasty for AMI: women	Coronary angioplasty for AMI: RR (95% CI)	
20-24	3.4	0.9	3.62 (3.23 to 4.06)	1.9	0.6	3.11 (2.70 to 3.59)	0.2	0.0	3.09 (1.87 to 5.11)	
25-29	6.1	1.9	3.25 (3.00 to 3.52)	3.3	1.1	3.18 (2.85 to 3.54)	0.7	0.2	3.32 (2.63 to 4.19)	
30-34	12.2	4.1	2.96 (2.80 to 3.12)	6.2	2.0	3.11 (2.87 to 3.37)	2.5	0.8	3.26 (2.87 to 3.71)	
35-39	24.8	9.8	2.52 (2.43 to 2.61)	11.3	4.2	2.70 (2.55 to 2.85)	7.1	2.3	3.06 (2.85 to 3.29)	
40-44	52.7	22.3	2.36 (2.30 to 2.42)	21.0	8.6	2.44 (2.34 to 2.53)	18.0	6.1	2.93 (2.80 to 3.06)	
45-49	101.8	43.7	2.33 (2.28 to 2.37)	38.4	14.9	2.58 (2.50 to 2.66)	37.8	12.9	2.94 (2.84 to 3.03)	
50-54	175.2	71.6	2.44 (2.41 to 2.48)	64.6	23.6	2.74 (2.67 to 2.81)	68.0	23.1	2.95 (2.87 to 3.02)	
55-59	266.5	111.0	2.40 (2.37 to 2.43)	103.1	38.7	2.66 (2.61 to 2.72)	102.3	37.4	2.73 (2.67 to 2.79)	
60-64	362.9	161.5	2.25 (2.22 to 2.27)	153.7	65.0	2.37 (2.32 to 2.41)	135.9	55.5	2.45 (2.40 to 2.50)	
65-69	439.1	206.4	2.13 (2.10 to 2.15)	218.5	102.4	2.13 (2.10 to 2.17)	156.2	66.0	2.37 (2.32 to 2.41)	
70-74	477.7	246.0	1.94 (1.92 to 1.97)	294.2	153.9	1.91 (1.88 to 1.94)	160.4	75.8	2.12 (2.07 to 2.16)	
75-79	489.0	277.5	1.76 (1.74 to 1.79)	390.2	227.4	1.72 (1.69 to 1.74)	153.8	80.1	1.92 (1.87 to 1.97)	
80+	450.0	286.9	1.57 (1.55 to 1.59)	679.8	500.2	1.36 (1.34 to 1.37)	120.4	68.9	1.75 (1.70 to 1.80)	

**Figure 2:**
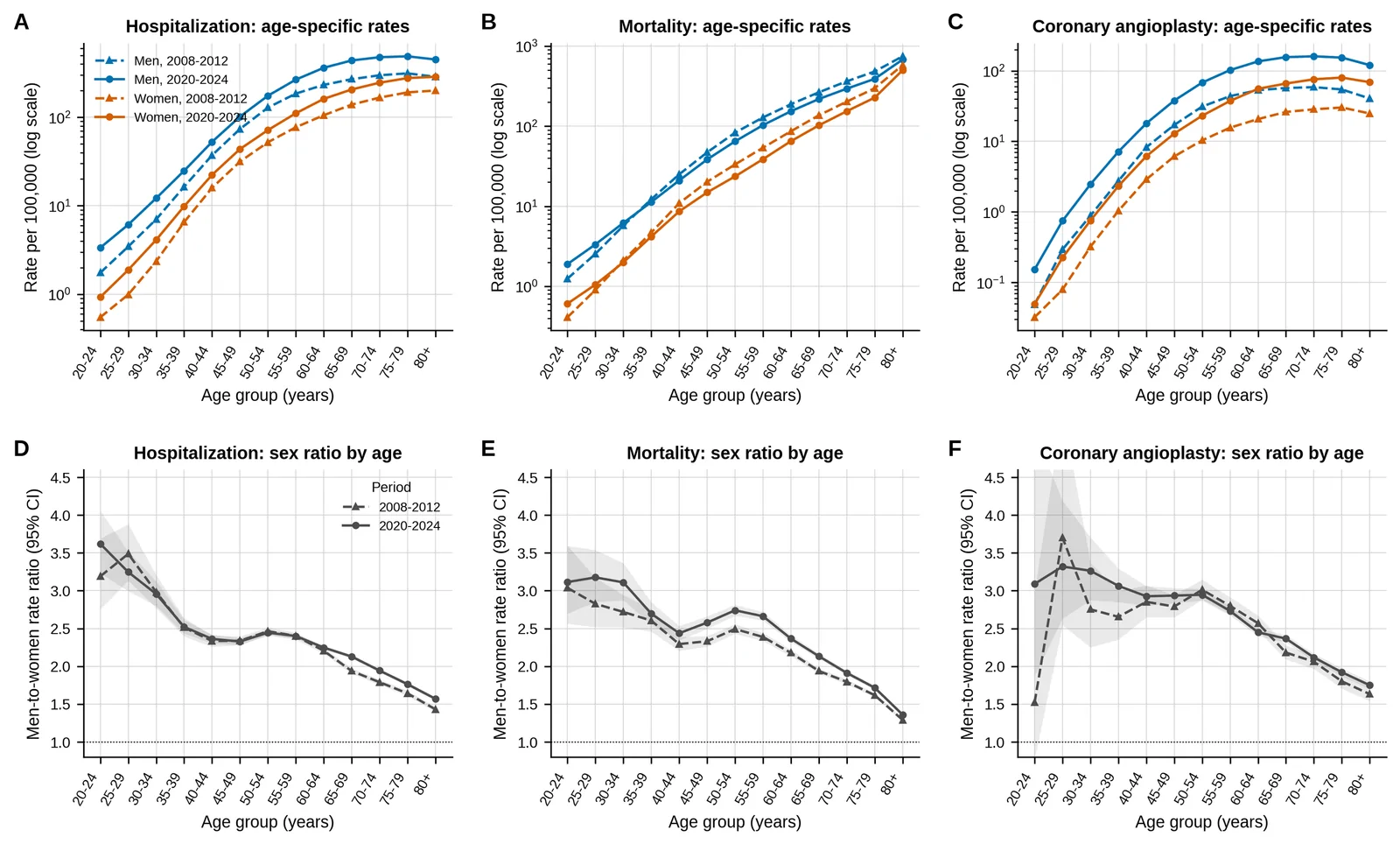
Age-specific rates and men-to-women rate ratios in two periods in Brazil Panels A to C show age-specific rates per 100,000 population per year by five-year age group (logarithmic scale) for men (blue) and women (orange), pooled over 2008-2012 (dashed lines, triangles) and 2020-2024 (solid lines, circles). Panels D to F show the corresponding men-to-women rate ratios with 95% confidence intervals in the same two periods; the dotted line marks a ratio of 1. Age groups below 20 years contributed few events and are omitted. Rates were standardized by age and sex to the 2022 Brazilian Demographic Census [19]; denominators are the Brazilian Institute of Geography and Statistics (IBGE) population projections, 2024 revision [18]. Sources: Hospital Information System of the Unified Health System (SIH/SUS) [14] for hospitalization and angioplasty; Mortality Information System (SIM) [15] for mortality; International Classification of Diseases, 10th Revision (ICD-10) I21-I23 [16].

In-hospital case fatality

Among patients admitted with infarction, in-hospital deaths numbered 4690 of 39,832 admissions (11.8%) in men and 3909 of 23,050 (17.0%) in women in 2008, and 7859 of 116,097 (6.8%) and 6176 of 66,479 (9.3%) in 2024. Age-standardized case fatality fell from 12.9% to 7.0% in men and from 16.1% to 8.3% in women (annual percent change, -3.66% (95% CI, -4.47 to -2.85) and -3.78% (-4.49 to -3.07)), with a sharp acceleration after 2021 (men, -7.29% per year; women, -7.79%) (Table 3, Figure 3). After adjustment for age and year, men had a 16% lower risk of dying in hospital than women (relative risk, 0.84; 95% CI, 0.83 to 0.85), and the difference did not attenuate over time (p=0.89). Among admissions with angioplasty the pattern was the same at a lower level: in-hospital deaths numbered 391 of 7441 (5.3%) in men and 278 of 3435 (8.1%) in women in 2008, and 1448 of 43,399 (3.3%) and 1124 of 22,280 (5.0%) in 2024, and age-standardized case fatality fell from 5.9% to 3.3% in men and from 7.9% to 4.3% in women (relative risk, 0.77; 95% CI, 0.76 to 0.79; p=0.74 for change over time) (Figure 4). Women had higher case fatality in every year and in every age group from 40 years onward, for example 3242 of 43,010 (7.5%) vs. 5272 of 83,357 (6.3%) at 60 to 64 years and 8466 of 39,672 (21.3%) vs. 7459 of 38,523 (19.4%) at 80 years or older in 2020-2024 (Figure 5). Because admissions grew faster than case fatality fell, the number of in-hospital deaths rose from 8599 in 2008 to 14,035 in 2024.

**Figure 3:**
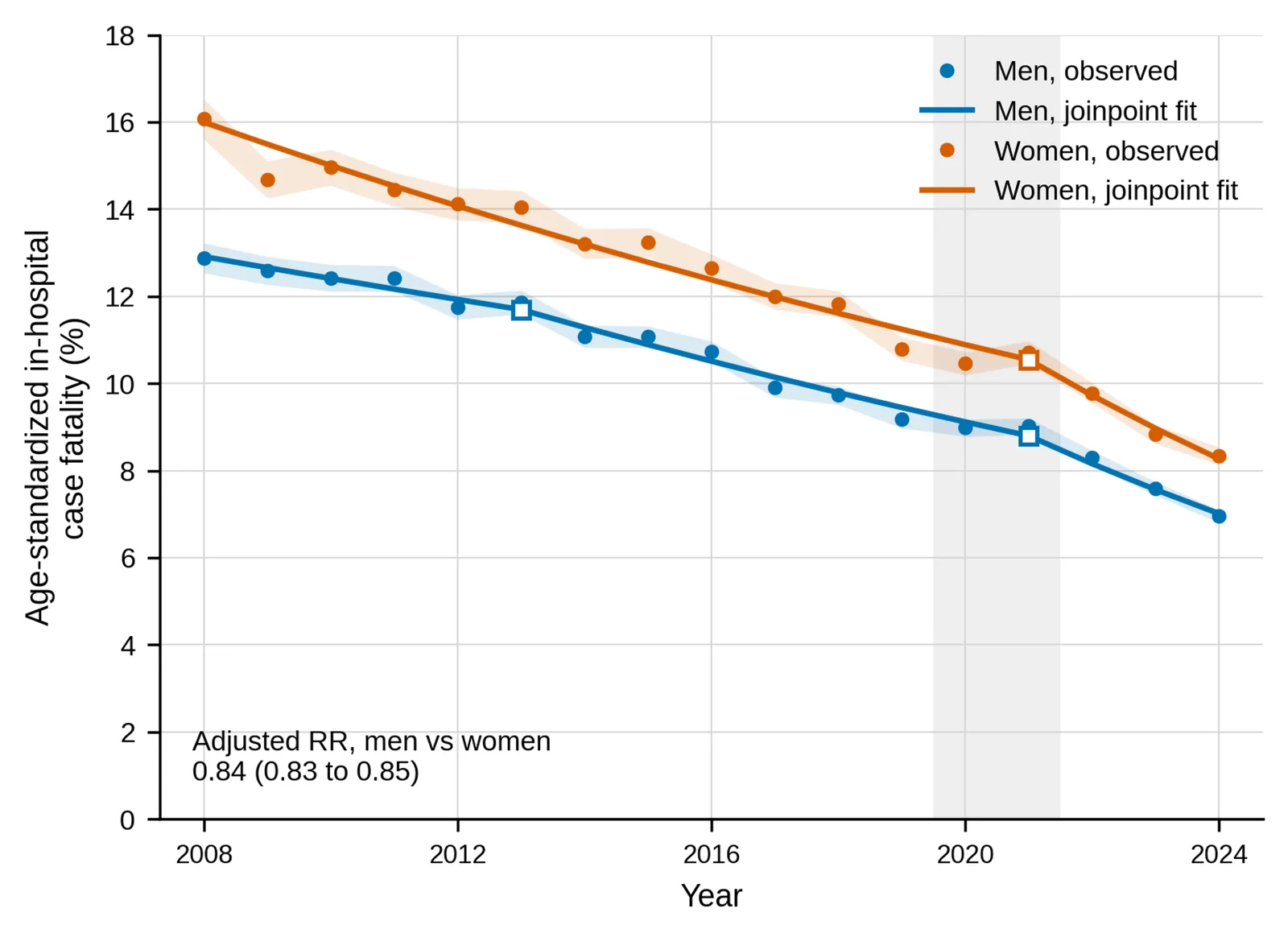
In-hospital case fatality among patients admitted for acute myocardial infarction by sex in Brazil, 2008-2024 Annual in-hospital case fatality, standardized to the age distribution of all acute myocardial infarction admissions over 2008-2024, in men (blue) and women (orange), with 95% confidence bands, observed values as points, joinpoint fits as solid lines, and joinpoints as open squares. The age- and year-adjusted relative risk of in-hospital death in men versus women, among those admitted, is inset. The shaded band marks the COVID-19 pandemic years 2020-2021. Sources: Hospital Information System of the Unified Health System (SIH/SUS) [14] for hospitalization and angioplasty; International Classification of Diseases, 10th Revision (ICD-10) I21-I23 [16].

**Figure 4:**
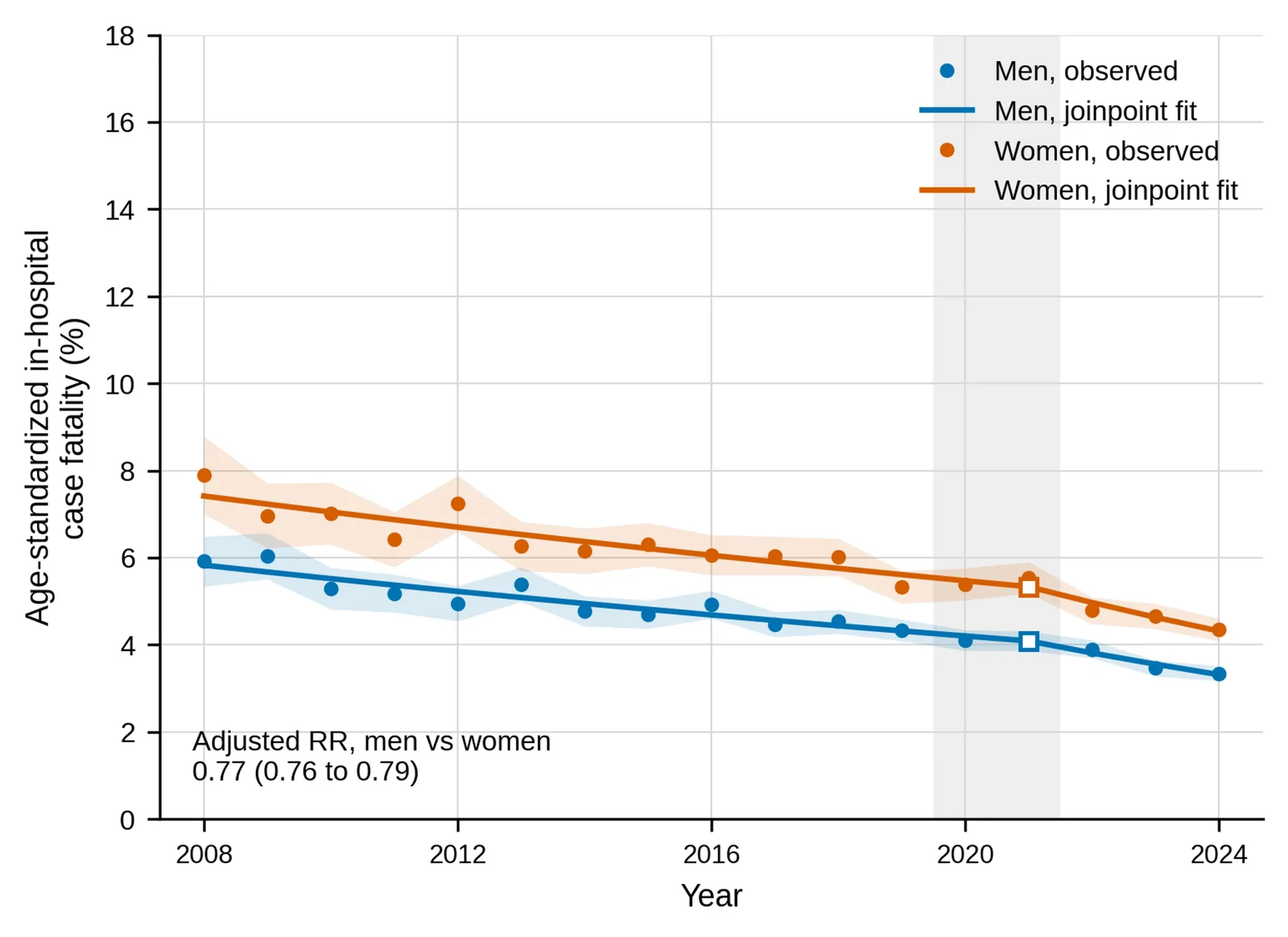
In-hospital case fatality among admissions with coronary angioplasty for acute myocardial infarction by sex the Brazil, 2008-2024 Plotted and standardized as in Figure 3, restricted to admissions in which one of six unified procedure schedule of the public system (SIGTAP) percutaneous coronary intervention codes was performed with acute myocardial infarction as the principal diagnosis [14,16,17]. The shaded band marks 2020-2021.

**Figure 5:**
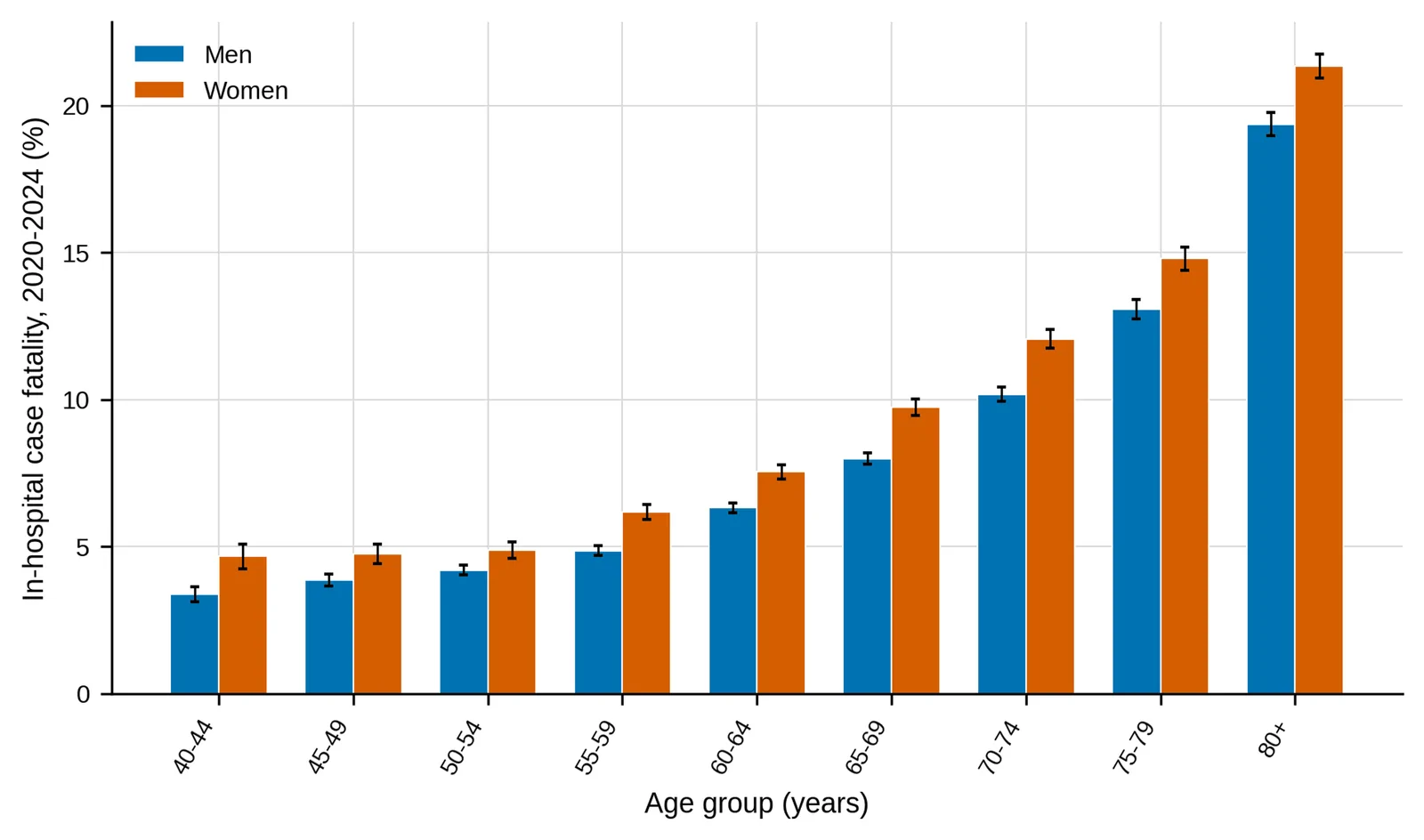
In-hospital case fatality by age group among patients admitted for acute myocardial infarction in Brazil, 2020-2024 Crude in-hospital case fatality in men (blue) and women (orange) by five-year age group, pooled over 2020-2024, with 95% confidence intervals. Age groups below 40 years contributed few deaths and are omitted. Sources: Hospital Information System of the Unified Health System (SIH/SUS) [14] for hospitalization and angioplasty; International Classification of Diseases, 10th Revision (ICD-10) I21-I23 [16].

Sensitivity analyses

Rates were standardized to the 2022 Census population (Appendix 1). The effect of standardization declined steadily, from +41.2% in 2008 to -0.9% in 2024 for hospitalization, as expected for an external standard applied to an aging population (complete annual series in Appendix 2-4). None of the six sensitivity analyses altered a conclusion (Appendix 5). Alternative standard populations changed the annual percent change by at most 0.1 percentage point; the younger 2008 population, which weights the ages of greatest male excess more heavily, raised the 2024 rate ratios by 0.03 to 0.04. The hospitalization trend in men ranged from +3.56% per year when the series began in 2010 to +3.88% when the pandemic years were excluded. Rescaling the denominators to the census count raised every rate by 3.7% and left every rate ratio unchanged. Defining angioplasty by infarction in any diagnostic field added 7890 admissions (1.5%) and left the adjusted rate ratio at 2.42.

## Discussion

Over 17 years, during which the Brazilian public system nearly doubled its rate of admission for acute myocardial infarction and almost quadrupled its rate of coronary angioplasty, while infarction mortality and in-hospital case fatality fell, the balance between men and women hardly changed. Men remained about twice as likely as women to be admitted and to undergo angioplasty, and one and a half times as likely to die of infarction. Among those admitted, men remained 16% less likely than women to die in hospital.

Read together, the three series describe a system that is reaching more patients. Rising admissions with falling mortality and falling case fatality is the pattern expected when infarctions that once ended in sudden death or went unrecognized now reach hospital, are recorded and are treated. The growth of angioplasty, more than twice as fast as that of admissions, supports this reading, as do national analyses reporting increases of 146% in emergency infarction admissions and 191% in primary angioplasty between 2008 and 2023 [8,12]. Those analyses also describe rising population-adjusted in-hospital mortality, which is consistent with our data: in-hospital deaths increased by 63% because admissions grew faster than case fatality declined. The abrupt fall in case fatality after 2021 coincided with renewed acceleration of angioplasty and may partly reflect that change in case mix, a possibility our data cannot test.

The persistently higher case fatality among women has the most direct clinical meaning, but it is a comparison among patients who were admitted. If women with suspected infarction are admitted under a different threshold than men, through atypical presentation, later recognition, or triage, the admitted groups are not alike, and part or all of the gap could reflect that selection rather than the care that follows. Administrative records capture the admission and its outcome, not the presentation, the delay, or the type of infarction, and cannot separate these explanations. With that caveat, the finding agrees with pooled trial data showing worse one-year outcomes in women after primary angioplasty despite similar infarct size [5], and with the Brazilian cohort in which middle-aged women had excess mortality after infarction [10]. It does not contradict registries that find no independent effect of sex [6,7]. Those studies begin at the catheterization laboratory and adjust for the clinical profile on arrival, whereas our estimate, adjusted only for age and year, includes everything that happens before. A difference that remained stable for 17 years is more plausibly a persistent upstream determinant than a biologic constant, in a system in which public patients already reach a hospital capable of intervention nearly three times later than insured patients [11].

The unchanged ratios admit three explanations. They may reflect a stable difference in disease occurrence, in which case a symmetric expansion of care should leave them untouched; the age gradient, from ratios near 3 in early adulthood to between 1.4 and 1.8 in the oldest group, fits the later onset of coronary disease in women [1,2]. They may also reflect less frequent revascularization of women. Because angioplasty admissions are a subset of infarction admissions, the higher rate ratio for angioplasty (2.42) than for admission (2.09) corresponds to a deficit of 3 to 5 percentage points in the proportion of women revascularized, present in every year. Whether that deficit reflects infarction type, anatomy, delay, or clinical choice cannot be determined without linked clinical data. Finally, men and women may use private care differently, which we could not measure. The upward drift in the mortality ratio, from 1.47 to 1.60, was the only hint of change, and we do not regard it as established: the interaction test, which accounts for overdispersion, gave p=0.10.

These findings add one observation to the international literature. Where sex disparities in ischemic heart disease mortality persist in Europe and the United States, they follow modifiable cardiometabolic and dietary risk [3], and in China the cardiovascular burden has fallen faster in women [4]. Brazil, during a period of rapid expansion, showed neither convergence nor divergence. Expanding access and reducing disparity appear to be separate objectives, and achieving the first did not deliver the second. For Brazil, this argues for routine sex-specific reporting of reperfusion and its timeliness, rather than of procedure volumes, and for attention to the prehospital pathway, where regional variation is documented [8,11], and our data are silent.

Limitations

The study includes every infarction admission and death recorded nationally over 17 years, with one period and one standardization scheme for all outcomes, but it has limitations. The unit of observation is the admission, not the patient, so transfers and readmissions are counted more than once; if men are transferred for intervention more often, the hospital ratios are inflated, although the mortality ratio is unaffected. Hospital data cover public care only, and sex differences in the use of private care would bias the hospital ratios in an unknown direction; the mortality data include every death regardless of payer. In-hospital case fatality misses deaths before arrival and after discharge, and it is conditional on admission. Denominators are projections that place the 2022 population 3.8% above the census count, which shifts the rates but not the ratios. Finally, without clinical data, we cannot distinguish ST-segment elevation from other infarction, adjust for coexisting conditions, or attribute the case-fatality gap to presentation, delay, or treatment; the relative risk of 0.84 describes a difference and does not estimate a causal effect of sex.

## Conclusions

In Brazil's public health system, the expansion of acute coronary care over the study period improved outcomes of myocardial infarction in both sexes without narrowing the gap between them, and women admitted with infarction remained more likely than men to die in hospital. The data rule out any large change in the disparity over the period, although they cannot exclude a slow one. Closing the gap will require measuring, by sex, what happens before and during the first hours of care, beginning with the timeliness of reperfusion.

## Appendices

**Table 5:** Appendix 1 - Standard population and standardization weights, 2022 Brazilian Census Resident population enumerated in the 2022 Brazilian Demographic Census, collapsed to 17 age groups [19]. Joint weights (age by sex) sum to 1 across all cells and are used for rates in both sexes combined; within-sex weights sum to 1 in each sex and are used for sex-specific rates, so that the standardized rate for both sexes equals the population-weighted sum of the two sex-specific rates. The same standard was applied to the three outcomes.

Age group (years)	Men, n	Women, n	Both sexes, n	Joint weight, men	Joint weight, women	Within-sex weight, men	Within-sex weight, women
0-4	6,461,689	6,243,171	12,704,860	0.03182	0.03074	0.06558	0.05972
5-9	7,011,282	6,738,158	13,749,440	0.03452	0.03318	0.07116	0.06445
10-14	6,992,746	6,682,215	13,674,961	0.03443	0.03290	0.07097	0.06392
15-19	7,317,515	7,058,427	14,375,942	0.03603	0.03476	0.07427	0.06751
20-24	7,767,306	7,699,157	15,466,463	0.03825	0.03791	0.07883	0.07364
25-29	7,627,458	7,842,265	15,469,723	0.03756	0.03862	0.07741	0.07501
30-34	7,537,285	7,935,832	15,473,117	0.03711	0.03908	0.07650	0.07591
35-39	7,827,333	8,345,458	16,172,791	0.03854	0.04109	0.07944	0.07982
40-44	7,781,059	8,291,111	16,072,170	0.03832	0.04083	0.07897	0.07930
45-49	6,549,109	7,091,003	13,640,112	0.03225	0.03492	0.06647	0.06783
50-54	6,014,391	6,584,190	12,598,581	0.02962	0.03242	0.06104	0.06298
55-59	5,419,505	6,149,601	11,569,106	0.02669	0.03028	0.05500	0.05882
60-64	4,605,834	5,338,555	9,944,389	0.02268	0.02629	0.04674	0.05106
65-69	3,588,052	4,288,180	7,876,232	0.01767	0.02112	0.03641	0.04102
70-74	2,615,350	3,243,186	5,858,536	0.01288	0.01597	0.02654	0.03102
75-79	1,657,786	2,189,593	3,847,379	0.00816	0.01078	0.01682	0.02094
80+	1,758,731	2,828,223	4,586,954	0.00866	0.01393	0.01785	0.02705
Total	98,532,431	104,548,325	203,080,756	0.48519	0.51481	1.00000	1.00000

**Table 6:** Appendix 2 - Admissions for acute myocardial infarction: annual counts, population, crude and age-standardized rates by sex, with in-hospital deaths and standardized case fatality in Brazil, 2008-2024 Source: Hospital Information System of the Unified Health System (SIH/SUS) [14]; ICD-10 I21-I23 as principal diagnosis [16]. Rates per 100,000 population; denominators are IBGE population projections, 2024 revision [18]; rates directly standardized by age and sex to the 2022 Census [19] (Supplementary Table 5). "Standardized vs. crude" is the percentage difference between the standardized and the crude rate; its steady decline over the period is the expected behavior of an external standard applied to an aging population. Standardized case fatality is directly standardized to the age distribution of all admissions for this outcome over 2008-2024, both sexes, and refers to patients who were admitted.

Year	Sex	Admissions, n	Population	Crude rate	Standardized rate (95% CI)	Standardized vs. crude, %	In-hospital deaths, n	Standardized case fatality, % (95% CI)
2008	Men	39,832	93,648,115	42.5	59.8 (59.2 to 60.3)	+40.5	4,690	12.88 (12.54 to 13.22)
	Women	23,050	97,687,081	23.6	33.8 (33.4 to 34.3)	+43.4	3,909	16.07 (15.60 to 16.53)
	Both sexes	62,882	191,335,196	32.9	46.4 (46.0 to 46.8)	+41.2	8,599	14.10 (13.83 to 14.38)
2009	Men	43,816	94,518,589	46.4	63.9 (63.3 to 64.5)	+37.9	5,039	12.59 (12.27 to 12.91)
	Women	25,602	98,655,447	26.0	36.4 (36.0 to 36.9)	+40.3	3,951	14.68 (14.26 to 15.11)
	Both sexes	69,418	193,174,036	35.9	49.8 (49.4 to 50.1)	+38.5	8,990	13.36 (13.11 to 13.62)
2010	Men	48,029	95,258,934	50.4	67.9 (67.3 to 68.6)	+34.8	5,426	12.42 (12.11 to 12.73)
	Women	27,514	99,490,395	27.7	38.0 (37.5 to 38.4)	+37.3	4,353	14.96 (14.55 to 15.37)
	Both sexes	75,543	194,749,329	38.8	52.5 (52.1 to 52.9)	+35.4	9,779	13.36 (13.12 to 13.60)
2011	Men	51,652	95,921,330	53.8	71.0 (70.4 to 71.6)	+31.8	5,881	12.41 (12.11 to 12.70)
	Women	29,665	100,237,280	29.6	39.6 (39.2 to 40.1)	+33.9	4,575	14.45 (14.07 to 14.84)
	Both sexes	81,317	196,158,610	41.5	54.8 (54.5 to 55.2)	+32.3	10,456	13.20 (12.97 to 13.43)
2012	Men	54,403	96,643,211	56.3	72.7 (72.1 to 73.3)	+29.1	5,873	11.75 (11.47 to 12.03)
	Women	31,422	101,027,409	31.1	40.7 (40.2 to 41.1)	+30.8	4,764	14.12 (13.74 to 14.49)
	Both sexes	85,825	197,670,620	43.4	56.2 (55.8 to 56.6)	+29.4	10,637	12.66 (12.44 to 12.88)
2013	Men	55,594	97,392,749	57.1	72.2 (71.5 to 72.8)	+26.4	6,120	11.87 (11.59 to 12.14)
	Women	31,986	101,833,953	31.4	40.2 (39.7 to 40.6)	+27.8	4,839	14.05 (13.68 to 14.42)
	Both sexes	87,580	199,226,702	44.0	55.7 (55.3 to 56.0)	+26.7	10,959	12.67 (12.45 to 12.89)
2014	Men	60,441	98,158,110	61.6	76.1 (75.5 to 76.7)	+23.6	6,235	11.08 (10.82 to 11.33)
	Women	34,904	102,653,021	34.0	42.4 (41.9 to 42.8)	+24.6	4,958	13.21 (12.86 to 13.55)
	Both sexes	95,345	200,811,131	47.5	58.8 (58.4 to 59.1)	+23.7	11,193	11.88 (11.68 to 12.08)
2015	Men	64,646	98,927,365	65.3	79.0 (78.4 to 79.6)	+20.9	6,714	11.07 (10.82 to 11.32)
	Women	37,447	103,476,277	36.2	44.0 (43.6 to 44.5)	+21.7	5,352	13.24 (12.91 to 13.57)
	Both sexes	102,093	202,403,642	50.4	61.0 (60.6 to 61.4)	+21.0	12,066	11.89 (11.70 to 12.09)
2016	Men	68,909	99,630,921	69.2	81.8 (81.2 to 82.4)	+18.3	6,927	10.73 (10.50 to 10.97)
	Women	39,673	104,241,004	38.1	45.2 (44.8 to 45.7)	+18.8	5,460	12.65 (12.33 to 12.96)
	Both sexes	108,582	203,871,925	53.3	63.0 (62.6 to 63.4)	+18.3	12,387	11.46 (11.27 to 11.64)
2017	Men	71,968	100,267,821	71.8	83.1 (82.5 to 83.7)	+15.7	6,726	9.90 (9.68 to 10.12)
	Women	41,339	104,943,736	39.4	45.6 (45.2 to 46.1)	+15.9	5,384	12.00 (11.70 to 12.30)
	Both sexes	113,307	205,211,557	55.2	63.8 (63.4 to 64.2)	+15.5	12,110	10.71 (10.53 to 10.89)
2018	Men	76,426	100,895,555	75.7	85.7 (85.1 to 86.3)	+13.2	6,998	9.73 (9.52 to 9.94)
	Women	43,649	105,633,483	41.3	46.7 (46.3 to 47.2)	+13.1	5,585	11.83 (11.53 to 12.12)
	Both sexes	120,075	206,529,038	58.1	65.7 (65.3 to 66.0)	+12.9	12,583	10.49 (10.32 to 10.66)
2019	Men	83,969	101,551,234	82.7	91.5 (90.8 to 92.1)	+10.6	7,324	9.17 (8.97 to 9.37)
	Women	48,239	106,348,865	45.4	50.1 (49.6 to 50.5)	+10.4	5,701	10.80 (10.53 to 11.06)
	Both sexes	132,208	207,900,099	63.6	70.1 (69.8 to 70.5)	+10.3	13,025	9.79 (9.63 to 9.94)
2020	Men	84,076	102,140,954	82.3	89.2 (88.6 to 89.8)	+8.3	7,144	8.98 (8.79 to 9.18)
	Women	47,222	107,023,935	44.1	47.6 (47.1 to 48.0)	+7.8	5,391	10.46 (10.19 to 10.72)
	Both sexes	131,298	209,164,889	62.8	67.8 (67.4 to 68.1)	+7.9	12,535	9.54 (9.38 to 9.69)
2021	Men	90,302	102,550,853	88.1	93.7 (93.1 to 94.3)	+6.4	7,755	9.02 (8.83 to 9.21)
	Women	51,583	107,552,789	48.0	50.6 (50.2 to 51.1)	+5.6	6,030	10.71 (10.46 to 10.97)
	Both sexes	141,885	210,103,642	67.5	71.5 (71.2 to 71.9)	+5.9	13,785	9.64 (9.49 to 9.79)
2022	Men	104,290	102,875,114	101.4	105.8 (105.2 to 106.5)	+4.4	8,348	8.29 (8.12 to 8.46)
	Women	59,821	107,987,869	55.4	57.2 (56.8 to 57.7)	+3.3	6,479	9.78 (9.55 to 10.01)
	Both sexes	164,111	210,862,983	77.8	80.8 (80.4 to 81.2)	+3.8	14,827	8.86 (8.72 to 8.99)
2023	Men	109,507	103,251,553	106.1	108.2 (107.6 to 108.9)	+2.1	8,025	7.59 (7.43 to 7.74)
	Women	63,600	108,443,605	58.6	59.1 (58.7 to 59.6)	+0.8	6,194	8.83 (8.62 to 9.05)
	Both sexes	173,107	211,695,158	81.8	83.0 (82.6 to 83.3)	+1.4	14,219	8.05 (7.92 to 8.17)
2024	Men	116,097	103,662,286	112.0	111.7 (111.1 to 112.3)	-0.3	7,859	6.95 (6.81 to 7.10)
	Women	66,479	108,921,464	61.0	60.0 (59.5 to 60.5)	-1.7	6,176	8.33 (8.13 to 8.54)
	Both sexes	182,576	212,583,750	85.9	85.1 (84.7 to 85.5)	-0.9	14,035	7.47 (7.36 to 7.59)

**Table 7:** Appendix 3 - Deaths from acute myocardial infarction: annual counts, population, crude and age-standardized rates by sex in Brazil, 2008-2024 Source: Mortality Information System (SIM) [15]; ICD-10 I21-I23 as underlying cause [16]. Rates per 100,000 population; denominators are Brazilian Institute of Geography and Statistics (IBGE) population projections, 2024 revision [18]; rates directly standardized by age and sex to the 2022 Census [19] (Supplementary Table 5). "Standardized vs. crude" is the percentage difference between the standardized and the crude rate; its steady decline over the period is the expected behavior of an external standard applied to an aging population.

Year	Sex	Deaths, n	Population	Crude rate	Standardized rate (95% CI)	Standardized vs. crude, %
2008	Men	44,438	93,648,115	47.5	69.0 (68.3 to 69.6)	+45.3
	Women	30,878	97,687,081	31.6	46.8 (46.3 to 47.3)	+48.0
	Both sexes	75,322	191,335,196	39.4	57.5 (57.1 to 58.0)	+46.2
2009	Men	45,034	94,518,589	47.6	67.8 (67.2 to 68.4)	+42.3
	Women	31,381	98,655,447	31.8	46.0 (45.5 to 46.5)	+44.7
	Both sexes	76,420	193,174,036	39.6	56.6 (56.2 to 57.0)	+43.0
2010	Men	47,018	95,258,934	49.4	68.7 (68.1 to 69.3)	+39.1
	Women	32,698	99,490,395	32.9	46.4 (45.8 to 46.9)	+41.0
	Both sexes	79,720	194,749,329	40.9	57.2 (56.8 to 57.6)	+39.7
2011	Men	48,777	95,921,330	50.9	69.1 (68.5 to 69.7)	+35.9
	Women	34,043	100,237,280	34.0	46.7 (46.2 to 47.2)	+37.4
	Both sexes	82,827	196,158,610	42.2	57.6 (57.2 to 58.0)	+36.3
2012	Men	49,542	96,643,211	51.3	68.0 (67.4 to 68.7)	+32.7
	Women	34,642	101,027,409	34.3	46.0 (45.5 to 46.5)	+34.1
	Both sexes	84,191	197,670,620	42.6	56.7 (56.3 to 57.1)	+33.1
2013	Men	50,512	97,392,749	51.9	67.2 (66.6 to 67.8)	+29.6
	Women	35,473	101,833,953	34.8	45.5 (45.0 to 46.0)	+30.7
	Both sexes	85,998	199,226,702	43.2	56.0 (55.7 to 56.4)	+29.8
2014	Men	51,410	98,158,110	52.4	66.2 (65.7 to 66.8)	+26.5
	Women	35,863	102,653,021	34.9	44.5 (44.1 to 45.0)	+27.4
	Both sexes	87,291	200,811,131	43.5	55.1 (54.7 to 55.4)	+26.7
2015	Men	53,413	98,927,365	54.0	66.7 (66.1 to 67.3)	+23.6
	Women	37,489	103,476,277	36.2	45.0 (44.5 to 45.5)	+24.2
	Both sexes	90,911	202,403,642	44.9	55.5 (55.2 to 55.9)	+23.6
2016	Men	55,663	99,630,921	55.9	67.4 (66.8 to 68.0)	+20.6
	Women	38,592	104,241,004	37.0	44.8 (44.3 to 45.2)	+20.9
	Both sexes	94,262	203,871,925	46.2	55.7 (55.4 to 56.1)	+20.6
2017	Men	54,726	100,267,821	54.6	64.2 (63.7 to 64.8)	+17.7
	Women	38,057	104,943,736	36.3	42.7 (42.3 to 43.2)	+17.8
	Both sexes	92,789	205,211,557	45.2	53.2 (52.8 to 53.5)	+17.6
2018	Men	54,984	100,895,555	54.5	62.5 (62.0 to 63.0)	+14.7
	Women	38,441	105,633,483	36.4	41.7 (41.3 to 42.1)	+14.6
	Both sexes	93,430	206,529,038	45.2	51.8 (51.5 to 52.1)	+14.5
2019	Men	56,630	101,551,234	55.8	62.4 (61.9 to 62.9)	+11.9
	Women	39,062	106,348,865	36.7	41.0 (40.6 to 41.4)	+11.5
	Both sexes	95,697	207,900,099	46.0	51.4 (51.0 to 51.7)	+11.6
2020	Men	53,989	102,140,954	52.9	57.8 (57.3 to 58.3)	+9.3
	Women	36,579	107,023,935	34.2	37.2 (36.8 to 37.6)	+8.8
	Both sexes	90,571	209,164,889	43.3	47.2 (46.9 to 47.5)	+9.0
2021	Men	56,599	102,550,853	55.2	59.2 (58.7 to 59.7)	+7.3
	Women	39,261	107,552,789	36.5	38.8 (38.5 to 39.2)	+6.4
	Both sexes	95,867	210,103,642	45.6	48.7 (48.4 to 49.0)	+6.8
2022	Men	58,263	102,875,114	56.6	59.5 (59.0 to 60.0)	+5.1
	Women	39,833	107,987,869	36.9	38.4 (38.0 to 38.8)	+4.1
	Both sexes	98,099	210,862,983	46.5	48.6 (48.3 to 48.9)	+4.5
2023	Men	56,444	103,251,553	54.7	56.0 (55.5 to 56.4)	+2.4
	Women	37,679	108,443,605	34.7	35.2 (34.8 to 35.6)	+1.3
	Both sexes	94,127	211,695,158	44.5	45.3 (45.0 to 45.6)	+1.8
2024	Men	56,621	103,662,286	54.6	54.4 (54.0 to 54.9)	-0.3
	Women	37,750	108,921,464	34.7	34.1 (33.8 to 34.4)	-1.6
	Both sexes	94,374	212,583,750	44.4	44.0 (43.7 to 44.3)	-0.9

**Table 8:** Appendix 4 - Admissions for acute myocardial infarction with coronary angioplasty: annual counts, population, crude and age-standardized rates by sex, with in-hospital deaths and standardized case fatality, Brazil, 2008-2024 Source: SIH/SUS [14]; six SIGTAP percutaneous coronary intervention codes [17] with ICD-10 I21-I23 as principal diagnosis [16]. Rates per 100,000 population; denominators are IBGE population projections, 2024 revision [18]; rates directly standardized by age and sex to the 2022 Census [19] (Supplementary Table S1). "Standardized vs. crude" is the percentage difference between the standardized and the crude rate; its steady decline over the period is the expected behaviour of an external standard applied to an ageing population. CI, confidence interval (Poisson approximation). Standardized case fatality is directly standardized to the age distribution of all admissions for this outcome over 2008-2024, both sexes, and refers to patients who were admitted.

Year	Sex	Admissions, n	Population	Crude rate	Standardized rate (95% CI)	Standardized vs. crude, %	In-hospital deaths, n	Standardized case fatality, % (95% CI)
2008	Men	7,441	93,648,115	7.9	11.1 (10.9 to 11.4)	+40.2	391	5.92 (5.34 to 6.49)
	Women	3,435	97,687,081	3.5	5.0 (4.9 to 5.2)	+43.6	278	7.90 (7.00 to 8.79)
	Both sexes	10,876	191,335,196	5.7	8.0 (7.9 to 8.2)	+40.8	669	6.53 (6.06 to 7.01)
2009	Men	8,880	94,518,589	9.4	12.9 (12.7 to 13.2)	+37.8	473	6.04 (5.51 to 6.57)
	Women	4,345	98,655,447	4.4	6.2 (6.0 to 6.4)	+40.6	319	6.96 (6.22 to 7.71)
	Both sexes	13,225	193,174,036	6.8	9.5 (9.3 to 9.6)	+38.3	792	6.28 (5.86 to 6.70)
2010	Men	9,761	95,258,934	10.2	13.8 (13.5 to 14.0)	+34.3	457	5.29 (4.82 to 5.77)
	Women	4,771	99,490,395	4.8	6.5 (6.4 to 6.7)	+36.6	354	7.02 (6.31 to 7.73)
	Both sexes	14,532	194,749,329	7.5	10.0 (9.9 to 10.2)	+34.6	811	5.92 (5.52 to 6.31)
2011	Men	11,333	95,921,330	11.8	15.5 (15.3 to 15.8)	+31.6	521	5.18 (4.75 to 5.61)
	Women	5,561	100,237,280	5.5	7.4 (7.2 to 7.6)	+33.6	372	6.42 (5.78 to 7.05)
	Both sexes	16,894	196,158,610	8.6	11.4 (11.2 to 11.5)	+31.9	893	5.56 (5.21 to 5.91)
2012	Men	12,731	96,643,211	13.2	17.0 (16.7 to 17.3)	+28.8	558	4.95 (4.55 to 5.35)
	Women	6,060	101,027,409	6.0	7.8 (7.6 to 8.0)	+30.2	462	7.24 (6.60 to 7.88)
	Both sexes	18,791	197,670,620	9.5	12.3 (12.1 to 12.4)	+28.9	1,020	5.74 (5.40 to 6.07)
2013	Men	14,042	97,392,749	14.4	18.1 (17.8 to 18.4)	+25.8	674	5.39 (4.99 to 5.78)
	Women	6,585	101,833,953	6.5	8.2 (8.0 to 8.4)	+27.5	445	6.26 (5.70 to 6.83)
	Both sexes	20,627	199,226,702	10.4	13.0 (12.9 to 13.2)	+26.0	1,119	5.70 (5.38 to 6.02)
2014	Men	16,027	98,158,110	16.3	20.1 (19.8 to 20.4)	+23.2	700	4.77 (4.43 to 5.12)
	Women	7,781	102,653,021	7.6	9.4 (9.2 to 9.6)	+24.2	510	6.15 (5.63 to 6.68)
	Both sexes	23,808	200,811,131	11.9	14.6 (14.4 to 14.8)	+23.2	1,210	5.25 (4.97 to 5.54)
2015	Men	17,754	98,927,365	17.9	21.6 (21.3 to 22.0)	+20.6	772	4.70 (4.38 to 5.02)
	Women	8,594	103,476,277	8.3	10.1 (9.9 to 10.3)	+21.4	587	6.31 (5.81 to 6.80)
	Both sexes	26,348	202,403,642	13.0	15.7 (15.5 to 15.9)	+20.5	1,359	5.30 (5.03 to 5.57)
2016	Men	19,446	99,630,921	19.5	23.0 (22.7 to 23.3)	+17.9	880	4.92 (4.61 to 5.24)
	Women	9,582	104,241,004	9.2	10.9 (10.7 to 11.1)	+18.4	630	6.06 (5.60 to 6.52)
	Both sexes	29,028	203,871,925	14.2	16.8 (16.6 to 17.0)	+17.8	1,510	5.34 (5.08 to 5.59)
2017	Men	21,192	100,267,821	21.1	24.4 (24.1 to 24.7)	+15.4	890	4.47 (4.18 to 4.76)
	Women	10,343	104,943,736	9.9	11.4 (11.2 to 11.6)	+15.8	688	6.04 (5.60 to 6.49)
	Both sexes	31,535	205,211,557	15.4	17.7 (17.5 to 17.9)	+15.2	1,578	5.06 (4.82 to 5.30)
2018	Men	23,383	100,895,555	23.2	26.2 (25.9 to 26.5)	+13.0	992	4.53 (4.26 to 4.81)
	Women	11,189	105,633,483	10.6	12.0 (11.8 to 12.2)	+13.1	728	6.01 (5.59 to 6.44)
	Both sexes	34,572	206,529,038	16.7	18.9 (18.7 to 19.1)	+12.8	1,720	5.03 (4.80 to 5.26)
2019	Men	26,694	101,551,234	26.3	29.1 (28.7 to 29.4)	+10.6	1,100	4.34 (4.09 to 4.59)
	Women	12,971	106,348,865	12.2	13.5 (13.2 to 13.7)	+10.4	766	5.32 (4.95 to 5.70)
	Both sexes	39,665	207,900,099	19.1	21.0 (20.8 to 21.2)	+10.2	1,866	4.68 (4.47 to 4.88)
2020	Men	28,767	102,140,954	28.2	30.5 (30.2 to 30.9)	+8.3	1,117	4.10 (3.87 to 4.34)
	Women	13,662	107,023,935	12.8	13.8 (13.5 to 14.0)	+7.8	812	5.39 (5.03 to 5.76)
	Both sexes	42,429	209,164,889	20.3	21.9 (21.7 to 22.1)	+7.9	1,929	4.53 (4.34 to 4.73)
2021	Men	30,633	102,550,853	29.9	31.8 (31.4 to 32.1)	+6.4	1,197	4.09 (3.86 to 4.32)
	Women	14,950	107,552,789	13.9	14.7 (14.4 to 14.9)	+5.6	908	5.54 (5.19 to 5.90)
	Both sexes	45,583	210,103,642	21.7	23.0 (22.8 to 23.2)	+5.9	2,105	4.59 (4.40 to 4.78)
2022	Men	34,789	102,875,114	33.8	35.3 (34.9 to 35.7)	+4.4	1,315	3.90 (3.69 to 4.11)
	Women	17,392	107,987,869	16.1	16.6 (16.4 to 16.9)	+3.2	935	4.79 (4.48 to 5.09)
	Both sexes	52,181	210,862,983	24.7	25.7 (25.5 to 25.9)	+3.8	2,250	4.22 (4.05 to 4.39)
2023	Men	38,203	103,251,553	37.0	37.8 (37.4 to 38.1)	+2.1	1,299	3.46 (3.28 to 3.65)
	Women	19,314	108,443,605	17.8	17.9 (17.7 to 18.2)	+0.8	1,009	4.66 (4.37 to 4.94)
	Both sexes	57,517	211,695,158	27.2	27.6 (27.3 to 27.8)	+1.5	2,308	3.87 (3.72 to 4.03)
2024	Men	43,399	103,662,286	41.9	41.8 (41.4 to 42.2)	-0.2	1,448	3.34 (3.17 to 3.51)
	Women	22,280	108,921,464	20.5	20.1 (19.8 to 20.4)	-1.7	1,124	4.35 (4.09 to 4.61)
	Both sexes	65,679	212,583,750	30.9	30.6 (30.4 to 30.8)	-0.9	2,572	3.72 (3.58 to 3.86)

**Table 9:** Appendix 5 - Sensitivity of trends and sex ratios to the standard population, start year, pandemic years, denominators and case definition in Brazil, 2008-2024 Annual percent change (APC) by Prais-Winsten regression of the logarithm of the annual age-standardized rate on calendar year; men-to-women (M:W) ratio of age-standardized rates. Each scenario was run through the same analytic code as the main analysis, changing one input. S-0 replaces the 2022 Census standard [19] by the IBGE projected population [18] of 2008 or summed over 2008-2024; the younger 2008 population gives more weight to the ages at which the male excess is largest and raises the ratios slightly. S-A starts the series after the 2008 transition to the unified procedure schedule [17]. S-B removes the two pandemic years from the trend fit. S-C multiplies every denominator by the constant that makes the 2022 projected population equal the census count; rates move and ratios do not. S-D defines coronary angioplasty by acute myocardial infarction recorded in any diagnostic field rather than as principal diagnosis. The first year is 2009 or 2010 in S-A. Sources: Sources: Hospital Information System of the Unified Health System (SIH/SUS) [14] for hospitalization and angioplasty; Mortality Information System (SIM) [15] for mortality; International Classification of Diseases, 10th Revision (ICD-10) I21-I23 [16].

Outcome	Scenario	APC, men, % (95% CI)	APC, women, % (95% CI)	Joinpoints, men	M:W ratio, first year	M:W ratio, 2024
Hospitalization	Main analysis, 2008-2024	+3.69 (+3.26 to +4.13)	+3.34 (+2.86 to +3.83)	2020	1.77	1.86
	S-0a Standard: projected population, 2008	+3.65 (+3.21 to +4.10)	+3.32 (+2.84 to +3.81)	2020	1.80	1.89
	S-0b Standard: projected population summed over 2008-2024	+3.67 (+3.23 to +4.11)	+3.33 (+2.85 to +3.82)	2020	1.77	1.86
	S-A1 Start in 2009	+3.60 (+3.13 to +4.07)	+3.24 (+2.70 to +3.78)	2020	1.76	1.86
	S-A2 Start in 2010	+3.56 (+3.02 to +4.10)	+3.24 (+2.63 to +3.85)	2020	1.79	1.86
	S-B Excluding 2020-2021	+3.88 (+3.47 to +4.28)	+3.53 (+3.13 to +3.93)	2011, 2017	1.77	1.86
	S-C Denominators rescaled to the 2022 Census (-3.7%)	+3.69 (+3.26 to +4.13)	+3.34 (+2.86 to +3.83)	2020	1.77	1.86
Mortality	Main analysis, 2008-2024	-1.44 (-1.91 to -0.98)	-1.92 (-2.56 to -1.27)	2016	1.47	1.60
	S-0a Standard: projected population, 2008	-1.43 (-1.87 to -0.98)	-1.92 (-2.55 to -1.29)	2016	1.50	1.64
	S-0b Standard: projected population summed over 2008-2024	-1.44 (-1.89 to -0.98)	-1.92 (-2.56 to -1.28)	2016	1.47	1.60
	S-A1 Start in 2009	-1.51 (-1.95 to -1.07)	-2.01 (-2.62 to -1.39)	2016	1.47	1.60
	S-A2 Start in 2010	-1.66 (-2.05 to -1.26)	-2.18 (-2.74 to -1.62)	2016	1.48	1.60
	S-B Excluding 2020-2021	-1.46 (-2.04 to -0.87)	-1.97 (-2.77 to -1.17)	2016	1.47	1.60
	S-C Denominators rescaled to the 2022 Census (-3.7%)	-1.44 (-1.91 to -0.98)	-1.92 (-2.56 to -1.27)	2016	1.47	1.60
Coronary angioplasty	Main analysis, 2008-2024	+8.22 (+7.68 to +8.77)	+8.17 (+7.64 to +8.70)	2014, 2021	2.21	2.08
	S-0a Standard: projected population, 2008	+8.14 (+7.59 to +8.70)	+8.10 (+7.55 to +8.66)	2014, 2021	2.26	2.12
	S-0b Standard: projected population summed over 2008-2024	+8.19 (+7.64 to +8.74)	+8.14 (+7.60 to +8.68)	2014, 2021	2.22	2.08
	S-A1 Start in 2009	+7.93 (+7.51 to +8.36)	+7.86 (+7.45 to +8.26)	2014, 2021	2.09	2.08
	S-A2 Start in 2010	+7.85 (+7.45 to +8.24)	+7.84 (+7.39 to +8.30)	2014, 2021	2.10	2.08
	S-B Excluding 2020-2021	+8.23 (+7.70 to +8.77)	+8.27 (+7.79 to +8.76)	2012	2.21	2.08
	S-C Denominators rescaled to the 2022 Census (-3.7%)	+8.22 (+7.68 to +8.77)	+8.17 (+7.64 to +8.70)	2014, 2021	2.21	2.08
	S-D Case definition: AMI in any diagnostic field	+8.33 (+7.77 to +8.89)	+8.29 (+7.76 to +8.81)	2012	2.21	2.08
